# Future Perspectives in Oxidative Stress in Trisomy 13 and 18 Evaluation

**DOI:** 10.3390/jcm11071787

**Published:** 2022-03-24

**Authors:** Angelika Buczyńska, Iwona Sidorkiewicz, Ahsan Hameed, Adam Jacek Krętowski, Monika Zbucka-Krętowska

**Affiliations:** 1 Clinical Research Centre, Medical University of Bialystok, 15-276 Bialystok, Poland; iwona.sidorkiewicz@umb.edu.pl (I.S.); ahsan.hameed@umb.edu.pl (A.H.); adamkretowski@wp.pl (A.J.K.); 2 Department of Endocrinology, Diabetology and Internal Medicine, Medical University of Bialystok, 15-276 Bialystok, Poland; 3 Department of Gynecological Endocrinology and Adolescent Gynecology, Medical University of Bialystok, 15-276 Bialystok, Poland

**Keywords:** oxidative stress, trisomy 18 syndrome, trisomy 13 syndrome

## Abstract

Autosomal aneuploidies are the most frequently occurring congenital abnormalities and are related to many metabolic disorders, hormonal dysfunctions, neurotransmitter abnormalities, and intellectual disabilities. Trisomies are generated by an error of chromosomal segregation during cell division. Accumulating evidence has shown that deregulated gene expression resulting from the triplication of chromosomes 13 and 18 is associated with many disturbed cellular processes. Moreover, a disturbed oxidative stress status may be implicated in the occurrence of fetal malformations. Therefore, a literature review was undertaken to provide novel insights into the evaluation of trisomy 13 (T13) and 18 (T18) pathogeneses, with a particular concern on the oxidative stress. Corresponding to the limited literature data focused on factors leading to T13 and T18 phenotype occurrence, the importance of oxidative stress evaluation in T13 and T18 could enable the determination of subsequent disturbed metabolic pathways, highlighting the related role of mitochondrial dysfunction or epigenetics. This review illustrates up-to-date T13 and T18 research and discusses the strengths, limitations, and possible directions for future studies. The progressive unification of trisomy-related research protocols might provide potential medical targets in the future along with the implementation of the foundation of modern prenatal medicine.

## 1. Introduction

Trisomy 13 (T13), resulting in Patau syndrome, is a chromosomal condition with a prevalence rate of 1/5000 to 1/20,000 [1,2,3]. Trisomy 18 (T18), causing Edwards syndrome, is another frequent autosomal aneuploidy after Trisomy 21 (T21), affecting 1/6000 to 1/8000 live-birth fetuses [2,4]. The most frequent mechanism responsible for the apparition of complete homogenous T13 occurrence is the complete triplication of chromosome 13, generally resulted from maternal nondisjunction in meiosis. Additionally, less frequently, T13 occurs as a result of an unbalanced Robertsonian translocation and mosaicism formation [5]. T18 occurs most frequently as a result of complete 18 trisomy due to a maternal meiotic nondisjunction, which is the most common form (94%) [6]. Mosaic trisomy 18 is the second cause corresponding to fewer than 5% of occurrences, and fewer than 2% of cases are caused by an additional copy of long arm chromosome 18q [7]. These chromosomal aberrations generate many congenital abnormalities such as heart defects, gastrointestinal defects, tracheoesophageal abnormalities, endocrine disorders, vision and hearing disorders, and limb and nervous system anomalies [8,9,10]. Following the complexity of existing comorbidities, numerical chromosomal aberration, such as T13 and T18 are one of the main causes of miscarriage or stillbirth [11]. However, along with improvements in clinical management, an increasing survival rate of patients with these syndromes has been reported [4,12,13,14,15,16,17].

Recently, a broad range of genetic diseases have been investigated for the implications with oxidative stress and mitochondrial dysfunction in their pathogenesis [18]. Moreover, a growing number of studies have recently demonstrated that oxidative stress formation results from trisomy occurrence [19,20,21,22,23] and was observed to be responsible for the T21 phenotype [24,25,26]. T13 and T18 are the most frequently autosomal chromosome aberrations, excluding T21, where the pathogenesis of this chromosomal aberration is largely known, and numerous studies have been conducted [5,7,27,28,29]. The pathogenic changes related to T13 and T18 may also be associated with oxidative stress with important causative genes being primarily involved in the redox balance regulation. Comprehensive studies concerning the evaluation of the trisomies’ pathomechanism could explain the development of some malformations and the importance of oxidative stress, which can lead to a better understanding of the effects of the occurrence of these trisomies [4,30,31]. Consequently, a literature review was undertaken to provide novel insights into trisomy 13 and 18 pathogeneses, with a particular emphasis on the effects of oxidative stress. We highlight that this study may not meet the standards of a conventional literature review. However, our review provides suggestions that support the development of prenatal medicine. Bearing in mind the limited literature data, this hypothesis was supported with investigations performed in the field of T21 oxidative stress described below.

## 2. Materials and Methods

**Figure 1:**
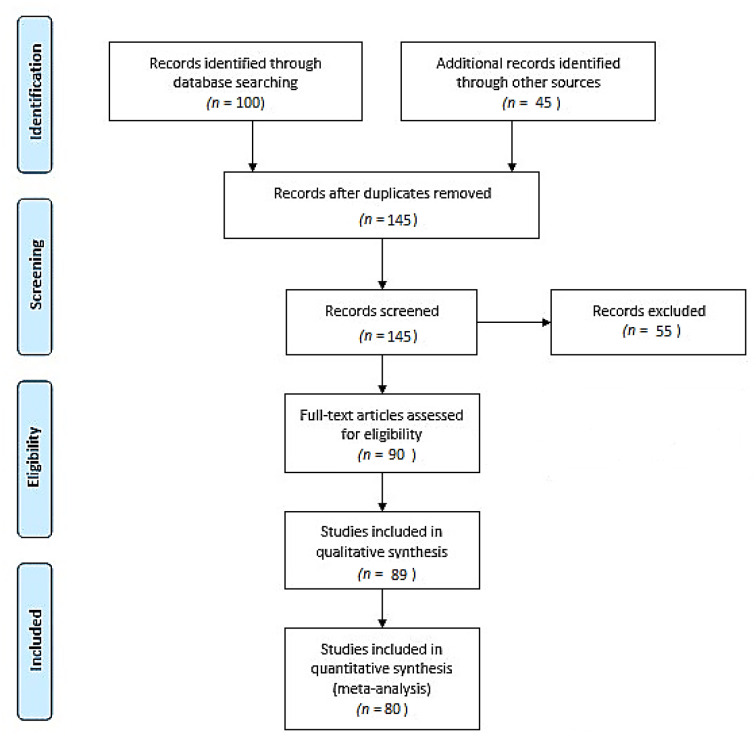
The flow diagram of the review process according to PRISMA guidelines [32,33].

The literature review was performed by searching different databases, including MEDLINE, PUBMED, and the Cochrane Library, according to the PRISMA and EQUATOR network guidelines [10,11,12,13], and was updated to December 2021, with no restrictions on the date of publication. This literature review followed the registered PROSPERO protocol (CRD42022298553) (Figure 1).

For this study, a review of the current literature regarding T13 and T18 evaluation was performed. The keywords used in the literature search were as follows: oxidative stress, trisomy 18, trisomy 13, trisomy 21, Edwards syndrome, Patau syndrome, Down syndrome, pathogenesis, antioxidant therapy, and potential therapy. Studies evaluating the latest reports based on pathogenesis, the impact of oxidative stress, and potential therapeutic target pathways were included. Moreover, the papers with inappropriate conclusions, study design, or irrelevant reporting were excluded during revision process.

## 3. Oxidative Stress: An Overview

All biological processes constitute a redox equilibrium, i.e., balanced oxidation and reduction reactions, to ensure convenient homeostasis [34]. Oxidative stress occurs due to a reduction in antioxidant defense caused by defects in the defense mechanisms and/or increased reactive oxygen species (ROS) synthesis [35]. ROS generation is directly associated with oxidized damage in biological components such as proteins, lipids, and DNA [36]. These deteriorations are mostly caused by O_2_^−^ (superoxide radical), OH^–^ (hydroxyl radical), and H_2_O_2_ (hydrogen peroxide) [37,38]. Recent studies have shown that mitochondrial dysfunction caused by oxidative stress plays an important role in neuronal damage and neurodegenerative diseases, which can be directly connected to the trisomic phenotype [36,39]. Mitochondrial respiratory chain complexes (MRCCs) play a key role in antioxidant defense by acting through the electron transport chain to oxidize hydrogen from the oxidation of organic acids with atomic oxygen to neutralize and expel hydrogen into water [36]. These complexes subsist as V cooperating units, which catalyze the phosphorylation of adenosine diphosphate (ADP) to adenosine triphosphate (ATP). Complex I is composed of nicotinamide adenine dinucleotide (NADH) coenzyme Q; complex II is composed of succinate dehydrogenase coenzyme Q; complex III is composed of coenzyme Q-cytochrome c reductase; complex IV is composed of cytochrome c oxidase; and complex V is composed of ATP synthase [40]. The MRCC is mostly exposed to oxidative stress through an increase in the possibility of oxidative damage caused to mitochondrial DNA (mtDNA), antioxidant proteins, and enzymes such as superoxide dismutase, catalase, glutathione peroxidase, and glutathione reductase in this complex, which may result in a subsequent additional increase in the intensity of the oxidative stress [41]. 

## 4. Previously Established T13 and T18 Pathogenesis—An Indicator for Oxidative Stress Testing

It has been shown that the composition of amniotic fluid, which is produced daily by the fetal urinary and respiratory systems using products of fetal skin keratinization, is similar to that of fetal plasma at the end of the second trimester [42,43]. Consequently, the concentrations of fetal proteins in second-trimester amniotic fluid are directly correlated with the concentrations in fetal serum, the analysis of which would facilitate the discovery of trisomy 13 and 18 pathogeneses [44,45,46,47]. Due to this fact, amniotic fluid appears to be the most useful material for analyzing abnormalities occurring in T13 and T18 fetal development [48]. 

One of the first studies, performed by Vrachnis, focused on resistin and leptin evaluations and showed that their deregulation may be implicated in T13 and T18 pathogeneses [31]. Resistin is a 12.5 kDa polypeptide secreted by adipocytes involved in insulin resistance development. Moreover, resistin is a potential marker of chronic inflammation associated with increased oxidative stress [49]. More interestingly, resistin can affect the function of nitric oxide synthase (eNOS) systems, resulting in a significant decrease in eNOS expression and nitric oxide (NO) production, thereby having antioxidative properties [31,50]. Leptin, a hormone released from the adipocytes, in addition to influencing the feeling of hunger, is also involved in antioxidant defense by decreasing ROS production [51]. 

Another study, performed by Hsu et al., aimed to evaluate T18 pathogenesis and was conducted on second-trimester amniotic fluid samples collected from six confirmed T18 pregnancies. The other six euploid pregnancies were enrolled as the control group [30]. The comparative proteomics analysis was performed using fluorescence-based two-dimensional difference gel electrophoresis (2D-DIGE) with matrix-assisted laser desorption/ionization time-of-flight mass spectrometry (MALDI-TOF/MS). The concentration of amniotic fluid apolipoprotein A1 (ApoA1) was increased in the T18-delivered samples compared to the euploid fluid samples [30]. Furthermore, the study demonstrated the deregulation of four proteins in T18 pregnancies: alpha-1-antitrypsin (A1AT, also known as serpin 1), vitamin D-binding protein (VDBP), insulin-like growth factor-binding protein 1 (IGFBP-1), and transthyretin (TTR) [30] (Table 1). ApoA1 is frequently used as a biomarker to predict cardiovascular diseases [45]. Its involvement in T18 could be associated with impaired lipid metabolism due to cardiovascular and neurological comorbidities during T18 early fetal development [46,52,53]. Moreover, the dysregulated *ApoA1* expression could also correspond to the oxidative damage observed in trisomy 21-based studies [22,54]. Concluding, ApoA1 plays a meaningful role in the pathogenesis of ES. A1AT is involved in the protection of neurons and glial cells from oxygen and glucose deprivation [55]. VDBP is an important component of many biochemical processes, including the transport of vitamin D and its metabolites, ensuring proper homeostasis. VDBP also controls essential proteins for proper bone metabolism, binding fatty acids, sequestering actin, and modulating oxidative and immune defenses [56,57]. IGFBP-1 serves as a carrier protein for insulin-like growth factors 1 and 2 (IGF1 and IGF2)—important determinants of fetal growth during pregnancy [58]. *TTR* gene mapped on 18q12.1 encodes a serum- and cerebrospinal fluid-binding protein for thyroxine and retinol implicated in fetal development [59]. Using a biological network analysis of T18 pathogenesis, Hsu et al. showed that the protein expression profile is associated with a lipid- and hormone-disturbed metabolic processes, improper immune response mechanisms, and cardiovascular comorbidities potentially connected to increased oxidative stress [30] (Table 1). 

## 5. Genetic Basis of the T13 and T18 Pathogeneses

There are several genes mapped on chromosomes 13 and 18 recognized as the players in the maintenance of redox balance [60]. Chromosome 13 mapping demonstrated the presence of genes associated with copper transport (ATPase copper transporting beta; *ATP7B*), tumor suppression (breast cancer 2; *BRCA2*), the inhibition of cell cycle processes, chromatin remodeling (retinoblastoma transcriptional corepressor 1; *RB1*), chromosome stability maintenance and regulations of chromosome segregation in mitosis (chromosome alignment-maintaining phosphoprotein 1; *CHAMP1*), and oxidative mitochondrial processes (mitochondrial intermediate peptidase; *MIPEP*), all of which are relevant in T13 pathogenesis [61,62,63,64]. The proper expression of the *ATP7B* gene is implicated in copper homeostasis, the deregulation of which may result in the development of many pathologies, especially those related to metabolic, cardiovascular and neurodegenerative diseases, and cancer [65]. Interestingly, the proper expression of *ATP7B* is crucial for mitochondrial protection against increased oxidative stress conditions, being an essential micronutrient for proper SOD-1 and mitochondrial complex IV activities [66]. In this case, this gene triplication may lead to an increased possibility of mtDNA mutation, resulting in subsequent oxidative stress disturbances according to the lack of mitochondrial antioxidant defense [67]. The *BRCA2* gene is also responsible for oxidative stress homeostasis; its overexpression correlates with increases in oxidative stress-restricted mtDNA replication, resulting in a disturbed mitochondrial oxidative balance [68]. Moreover, alterations in *MIPEP* expression, involved in oxidative phosphorylation (OXPHOS)-related protein maturation, may additionally indicate a connection between mitochondrial dysfunction and T13 development [64,69]. Moreover, the study performed by Renaudin et al. showed that *BRCA2* deficiency impairs ribonuclease H1 (RNaseH1) function, which is required to ensure mtDNA maintenance [68]. Interestingly, other genes, such as *RB1* and *CHAMP1*, are also related to oxidative-stress-related processes. It has been suggested that disturbances in *RB1* gene expression are involved in DNA damage sensor activity, forkhead box O (Foxo) transcription factors, and p38 mitogen-activated protein kinase processes, for which a disturbed expression affects cell-cycle progression, antioxidant capacity, mitochondrial mass, and cellular metabolism [70,71,72,73,74]. *CHAMP1* encodes a protein with a function in kinetochore–microtubule attachment and in the regulation of chromosome segregation. These properties are performed by their interaction and regulation of cell structure organization preceding mitosis, both of which are known to be important for proper fetal development [75,76]. Moreover, proper *MIPEP* expression is essential to maintain the normal level of mitochondrial sirtuin 3, which is considered a key regulator of oxidative stress by the deacetylation of the substrates involved in both ROS production and detoxification [77,78,79]. These mechanisms link oxidative stress to mitochondrial dysfunction and may be induced by the triplication of genes implicated in mitochondrial protective processes [80]. Referring to the fact that mitochondrial dysfunction is assumed to be one of the main T21-related symptoms [28,81], similar dysfunctions seem to be implicated in T13 development [61,68]. 

Furthermore, several important genes involved in intracellular cholesterol trafficking (Niemann–Pick C1 protein; *NPC1* gene), proper DNA transcription and signal transduction (mothers against decapentaplegic homolog; *SMAD*), and mitochondrial membrane function (ferrochelatase enzyme, coded by ferrochelatase; *FECH* gene) are mapped on chromosome 18 [82,83,84]. The *NPC1* gene encodes a crucial protein and affects the excitability of endosome and lysosome membranes, with characteristic mediation properties in intracellular cholesterol trafficking through cholesterol binding [82,85,86]. Interestingly, *NPC1* deficiency is related to neurodegenerative disease development due to oxidative damage. In this case, the *NPC1* gene’s correct expression is essential for oxidative stress balance [87]. Moreover, SMAD proteins are signal transducers and transcriptional modulators involved in multiple signaling pathways, such as cell growth, apoptosis, morphogenesis, and immune responses [83,88,89]. Research conducted by Xui et al. showed that *SMAD* overexpression results in increased oxidative stress and a reduction in cell viability with subsequent induction of apoptosis [90]. The *FECH* gene, which encodes the ferrochelatase enzyme, essential for the proper catalyzation of the insertion of the ferrous form of iron into the protoporphyrin heme synthesis pathway, is also related to oxidative stress homeostasis [84,91,92,93] (Table 2). 

The genes associated with additional chromosomes 13 and 18 are implicated in mitochondrial function and oxidative status. Therefore, a detailed evaluation of disturbed transcriptomic pathways related to T13 and T18 and the subsequent metabolic pathway disturbances may result in novel findings regarding trisomy-related abnormalities. Undoubtedly, studies may highlight deregulated pathways, and their detailed identification might become the basis for further research in T13 and T18 [47,94]. 

## 6. Uncoupling Oxidative Stress from the Pathogenesis of Trisomies: Future Perspectives

We strongly believe that comprehensive and extensive research can lead to a better understanding of trisomy-related comorbidities and the corresponding phenotypes [95]. In the following section, future perspectives are highlighted for T13 and T18 investigations in connection with T21 pathogenesis [47,55,96,97,98,99,100]. It is worth noting that an in vitro model for the study of trisomies other than T21 has not been reported in the literature. The unavailability of animal models has resulted in a subsequent lack of potential medical target evaluations. The combination of the current effective approaches shown during T21 research with additional relevant strategies proposed for T13 and T18 evaluations may provide life-saving treatments to the patients. 

### 6.1. Oxidative Stress and Lipid Peroxidation

The direct oxidative stress intensity measurement is complex following a short ROS residence time [101]. Due to the lack of methods by which to directly measure the oxidative processes, indirect investigations considering the levels of DNA/RNA damage, lipid peroxidation, and protein oxidation/nitration should be performed in this case [102]. Ischemia-modified albumin (IMA) generated by ROS has been found to be a sensitive and early biochemical marker of ischemic processes and is useful as an important marker of oxidative stress [103,104,105]. Importantly, neurons are highly sensitive to damage caused by oxidative stress exposure [106]. Increased oxidative stress may lead to neuroinflammation and cell death, resulting in progressive neurodegeneration [107]. Considering that reducing neurodegeneration is crucial for maintaining correct fetal development, aspects of oxidative stress influence, such as mitochondrial dysfunction and epigenetics, should be further evaluated in T13 and T18 studies [108]. In this case, other antioxidant proteins and activities of enzymes such as superoxide dismutase, catalase, xanthine oxidase, glutathione peroxidase, and glutathione reductase could be simply assessed in amniotic fluid samples using commercially available kits to evaluate the detailed associations between the oxidative stress and the phenotype of T13 and T18 trisomies [109,110,111,112,113]. To our knowledge, no adequate comparison has been performed for different oxidative stress biomarkers, mitochondrial dysfunction, and comorbidities. According to the literature data, deregulated lipid metabolism and the lipid peroxidation product (LPO) concentration have been observed as a result of mitochondrial dysfunction and elevated ROS formation [114]. LPOs, such as 8-isoprostane, 4-hydroxy-2-nonenal (4-HNE), and malondialdehyde (MDA) have been established as oxidative stress markers [35,115]. Moreover, they play a crucial role as signaling molecules in post-translational protein modification [115]. Furthermore, as highly reactive compounds, LPOs are also related to the generation of ROS and are capable of DNA and protein damage induction [116]. Fatty-acid-binding proteins (FABPs) are involved in the binding of free fatty acids, cholesterol, and retinoids, as well as in subsequent intracellular lipid transport [117,118,119]. Circulating FABP levels are physiologically low, but in pathological processes, their deregulation can be used to indicate tissue damage connected to improper epithelium function and ischemic processes [120,121]. Similarly, selected LPOs and FABPs with other oxidative stress markers could be evaluated to provide thorough information on lipid peroxidation and the involvement of oxidative stress in fetal development [122]. Moreover, recent studies highlight the interconnections between mitochondrial dysfunction and DS phenotype [95,123,124]. Following the promising results obtained in a T21 group based on an in vivo study, possible strategies to restore mitochondrial function and, therefore, to exert protective effects against the impact of increased oxidative stress on trisomy-associated pathologies can be discussed [28]. Thus, it can be assumed that oxidative stress is one of the leading causes of comorbidities in patients with T13 and T18 [55].

### 6.2. Mitochondrial Dysfunction

Mitochondrial dysfunction potentially constitutes a valuable component in T13 and T18 development based on the triplicated genes mapped on chromosomes 13 and 18 [5,7]. The most valuable function of mitochondria is OXPHOS, the oxygen-dependent production of ATP driven by MRCC. Notably, neurons are mostly dependent on OXPHOS, especially under oxidative stress conditions [114,125,126,127,128]. More importantly, NADPH oxidase is the main source of superoxide in first-trimester placentas [129]. A decrease in mitochondrial NADPH can indicate increased NADH oxidation, decreased NAD+ reduction, or increased NAD+ consumption, resulting in increased MRCC activity [126]. The correlation between NADPH measurements performed in maternal serum and amniotic fluid could describe the directions taken in oxidative stress development. Mitochondrial ribosomal protein L53 (MRPL53) is involved in the production of translational membrane proteins essential for OXPHOS [130]. Additionally, increased MRPL53 gene expression has been associated with the occurrence of orofacial clefting. [131,132]. The mitochondrial open reading frame of 12S rRNA-c (MOTS-c) was recently reported to regulate metabolic homeostasis with AMP-activated protein kinase (AMPK) activation, considered to be a supervisor of metabolic and mitochondrial oxidative stress homeostasis [133,134,135,136]. The importance of MOTS-c measurement during pregnancy was demonstrated by Wojciechowska et al. [137]. They showed an increase in the concentration of MOTS-c in the maternal blood and newborns of obese subjects and a corresponding decrease in the mothers and newborns in the group with hypothyroidism [137]. In this case, the disturbance in mitochondrial marker concentration, such as MOTS-c and MRPL53, may be one of the causes and an effect of an additionally disturbed energy metabolic rate, which could be involved in improper fetal development [137]. According to the fact that mitochondria also produce precursors for the synthesis of macromolecules such as DNA/RNA, proteins, and lipids, the complex evaluation of mitochondrial dysfunction during T13 and T18 development could describe an association between increased oxidative stress and related comorbidities [138,139].

### 6.3. Oxidative Stress Meets Epigenetics: An Implication in Trisomy Development

Oxidative stress conditions impair the function of nicotinamide adenine dinucleotide (NAD)-dependent deacetylases (HDACs) with a relevant sirtuin subgroup [140]. HDACs are involved in the epigenetic control of gene expression and cell cycling via the induction of G1-phase cell cycle arrest in cooperation with the p53 protein [141,142]. Histone deacetylases are responsible for increasing the positive charge of histone tails and stimulating high-affinity binding between the histones and DNA. Increased DNA binding condenses the DNA structure, inhibiting transcription [140,143,144,145]. Several studies have indicated that DNA methylation and histone deacetylation are reciprocally connected [146], resulting in the inhibition of transcription [147,148]. Global changes in methylation can be quantified by measuring the plasma levels of 5-methyl-2′-deoxycytidine. An imbalance between histone acetylation and deacetylation may cause inappropriate gene expression, was observed during T21 development and thus may have similar significance in other trisomies [140,141,143,149,150,151]. Based on the evaluation of T21 methylation processes, T21 development is associated with genome-wide perturbations in gene expression, which may contribute to a high frequency of health problems [143,145]. 

The sirtuin subfamily has also been linked to several oxidative-stress-related processes, such as mitochondrial dysfunction, gene transcription, the deacylation of histones, and DNA damage repair. Antioxidant processes are stimulated by the activation of various transcription factors [116,152,153]. SIRT1 and HDAC enzyme 1 are also involved in protein 53 (p53) activation [154]. The reregulation of p53 combined with oxidative stress development leads to the formation of oxidative DNA/RNA products such as 8-oxoguanine (8-oxoG) and 8-hydroxy-2-deoxy guanosine (8-OH-DG), which originate especially from mitochondrial DNA damage and related repair mechanisms, and can be quantified as indirect markers of oxidative-stress-related impairment [154,155]. An accurate analysis of the impacts of oxidative stress on SIRT1, HDAC enzymes, and p53 function with the quantification of DNA/RNA damage in T13 and T18 pregnancies could enable the detection of insufficient epigenetic pathways potentially leading to novel medical targets discovery. Considering the future possibilities of conducting in vitro/in vivo studies, the implementation of prenatal treatment could be introduced.

## 7. Perspectives

Omics data, obtained by applying advanced molecular biology techniques, could provide large-scale data that can be used to evaluate particularly significant pathways in the pathogenesis of trisomy development [156]. Prenatal diagnoses have witnessed significant progress; however, clinical management can be further improved, and possible medical treatment can be introduced [157,158]. The overexpression of genes mapped on chromosomes 13 and 18 leads to many congenital anomalies [159]. Notably, studies concerning T13 and T18 demonstrating metabolic changes closely related to oxidative stress have been performed [8,160]. Thus, it can be hypothesized that oxidative stress is one of the leading causes of comorbidities in patients with T13 and T18 [55]. As the pathogenic changes generated by trisomy are unknown, since the currently available methods and research models are insufficient, assessing the effects of trisomy, including the effects of oxidative stress on homeostasis, is of utmost importance [30,41]. Referring to the promising results of T21 pathogenesis evaluation, which establishes oxidative stress as one of the main disturbed pathways, similar studies should be conducted in other trisomies. 

Comprehensive research aimed at clarifying the relationship between transcription and methylation processes would enable further understanding of T13 and T18 [43]. A detailed evaluation of the influence of oxidative stress on cell-cycle processes could help reduce the occurrence rate of oxidative-stress-related disorders affecting a developing fetus [3]. 

Conceivably, the use of antioxidant nutrients to scavenge ROS may modulate congenital anomalies development in trisomic fetuses [27,161]. Unfortunately, prenatal treatments for trisomy-related fetal malformations have not yet been introduced despite numerous studies performed in T21 animal models [152,162,163,164]. It can be assumed that fetal brain development is affected by T13 and T18 and can be improved by inhibiting ROS activity at an early stage, resulting in similar outcomes to those in previous T21 studies [29,55,165,166,167]. To date, only in vivo animal therapeutic trials have been introduced [168]. Detailed T21 mouse model metabolic profiles showed oxidative stress (lipid peroxidation with protein carbonylation) and mitochondrial functional defects in the hippocampus and cortex, which resulted in neurobiological and cognitive T21 phenotypes [169,170]. TS mice supplemented with antioxidants, such as α-tocopherol and vitamin E, showed reduced oxidative stress and cholinergic neuron transmission degeneration, protected hippocampal morphology, and advanced spatial acting memory [165]. Similar results were obtained following melatonin supplementation [162]. Despite the promising results demonstrated in preclinical studies in the TS adult-stage mouse model, inconsistent research data have been reported regarding pathogenic changes induced by chromosomal aberration [166].

Due to the lack of research investigating the pathomechanism of defect development in cases of T13 and T18, we focused primarily on highlighting the directions for future research, emphasizing the importance of trisomy-related oxidative stress aspect and indicating the links to T21 research [30,55,60]. Comparable studies, such as those mentioned for T21, could also be performed in T13 and T18 groups to increase our knowledge regarding chromosomal aberration occurrence. Moreover, individuals receiving antioxidant supplementation showed significant improvements in cognitive functioning and the stabilization of cognitive decline [165]. These findings may allow the possibility of introducing prenatal treatments and can highlight many congenital anomalies resulting from chromosomal aberrations.

## 8. Conclusions

Despite the limitations in unraveling trisomy pathogenesis, oxidative stress has been suggested as a significant factor in T13 and T18 pathogeneses. The evaluation of oxidative stress-based disturbances in T13 and 18 may have a beneficial impact on prenatal management. Simultaneous pathogenesis profiling could increase the possibility of introducing prenatal treatment.

## Figures and Tables

**Table 1:** Disturbances in protein concentrations related to T13 and T18 pathogeneses [30,31].

Material	Protein	Full Name	Form of Dysregulation	Reference
Amniotic fluid T18 pregnancy	A1AT	alpha-1-antitrypsin	down	[30]
Amniotic fluid T18 pregnancy	ApoA	apolipoprotein A	up	[30]
Amniotic fluid T18 pregnancy	IGFBP-1	insulin-like growth factor-binding protein 1	down	[30]
Amniotic fluid T13 and T18 pregnancy	leptin	-	down	[31]
Amniotic fluid T13 and T18 pregnancy	resistin	-	down	[31]
Amniotic fluid T18 pregnancy	TTR	transthyretin	down	[30]
Amniotic fluid T18 pregnancy	VDBP	vitamin D binding protein	down	[30]

T13, trisomy 13; T18, trisomy 18.

**Table 2:** Gene expression related to T13 and T18 pathogeneses.

Gene Location	Gene	Full Name	Function
Chromosome 13	*ATP7B*	ATPase Copper Transporting Beta	copper transport
Chromosome 13	*BRCA2*	Breast Cancer 2	tumor suppression
Chromosome 13	*CHAMP1*	Chromosome Alignment-Maintaining Phosphoprotein 1	chromosome alignment maintenance with zinc finger protein regulations of chromosome segregation in mitosis
Chromosome 13	*MIPEP*	Mitochondrial Intermediate Peptidase	oxidative mitochondrial processes
Chromosome 13	*RB1*	Retinoblastoma Transcriptional Corepressor 1	inhibition of cell cycle processes, chromatin remodeling
Chromosome 18	*FECH*	Ferrochelatase	mitochondrial membrane function
Chromosome 18	*NPC1*	Niemann–Pick C1 Protein	intracellular cholesterol trafficking
Chromosome 18	*SMAD*	Mothers Against Decapentaplegic Homolog	transcription and signal transduction

## Data Availability

Not applicable.
